# Use of Molecular Logic Gates for the Tuning of Chemosensor Dynamic Range

**DOI:** 10.3390/molecules29184330

**Published:** 2024-09-12

**Authors:** Orhan Acikgoz, Christopher Abelt

**Affiliations:** Department of Chemistry, College of William and Mary, Williamsburg, VA 23185, USA; cjabel@wm.edu

**Keywords:** logic gates, chemosensors, fluorescence

## Abstract

Dynamic range is a crucial aspect in the development of fluorescent chemosensors. We aimed to address this issue using molecular logic gates. By creating an AND logic gate with two binding sites for the same type of ion, we increased the dynamic range of a sodium chemosensor while still using the same ionophore. Naphthalimide derivatives **1** and **2** were synthesized to test the plausibility of this application. Being an AND logic gate, the second molecule requires two Na^+^ ions, while molecule **1** requires a single ion for sensing. The application of this molecular logic gate is a useful method of altering the chemosensor range.

## 1. Introduction

Fluorescent chemosensors are powerful analytical tools that are used in the quantitative detection of ions, small neutral molecules, and some biological macromolecules. Since the 1980s, they have been synthesized and employed in various fields such as cell biology, medicine, environmental sciences, etc. [1,2,3]. Several challenges must be overcome for the synthesis of new chemosensors [4,5]. For example, the chemosensor must be selective toward the target compound and not respond to other ions or compounds in the solution. Fields such as cell biology have a demand for chemoselectivity, since Na^+^ and K^+^ are both crucial ions in cellular systems and a given analyte needs to be quantified without interference from other ions in the system [6]. Many other concerns such as solubility in aqueous solution, membrane permeability, high Stokes shift, etc. must be addressed for the development of chemosensors [7].

Another such challenge in this regard relates to the dynamic range of the chemosensor [6]. The dynamic range is the span of analyte concentrations over which the chemosensor is receptive. Concentrations that fall below the detection limit will not generate a fluorescence response. Conversely, changes in concentration that are outside of the dynamic range cannot be detected, since the chemosensors in solution would be saturated, and display maximum fluorescence. This range of concentration depends on the binding affinity between the ionophore and the analyte.

The concern of dynamic range creates a demand for the development of chemosensors that specifically match the relevant concentration. For example, the calcium concentration measured in an environmental study could be orders of magnitude higher than in a cell biology study. A great example of this problem was observed when Minta and Tsien developed a chemosensor for the detection of intracellular Na^+^ concentration [8]. The same chemosensor, however, could not be used in an extracellular study as biological cells tend to have lower sodium in their interior. The extracellular sodium concentration in this case was beyond the saturation point of the chemosensor.

In 1993, de Silva et al. developed the first molecular logic gate [9]. This probe served as an AND logic gate through the incorporation of a crown ether and a tertiary amine for the detection of H^+^ and Na^+^ ions. Unless both sites are occupied by the respective analyte, a fluorescence response will not be generated. Through the incorporation of multiple binding sites, molecules could be engineered to execute two-input logic functions. For a two-input logic gate, there are four different combinations of input: absence of both analytes, presence of one or the other analyte, and presence of both analytes. For each combination, an output may be absent or present. Since there are four combinations of input and two possible outputs, there exists a total of 16 (2^4^) two-input logic gates. Depending on the quenching mechanism between the ionophore and the fluorophore, chemosensors can behave like AND, OR, XOR, NAND, and such logic gates [10]. This behavior allowed for the detection of multiple ions in various ways. For example, Akkaya et al. developed a BODIPY-based AND chemosensor that displays fluorescence when both Hg^2+^ and Zn^2+^ are present in the solution [11].

**Figure 1:**
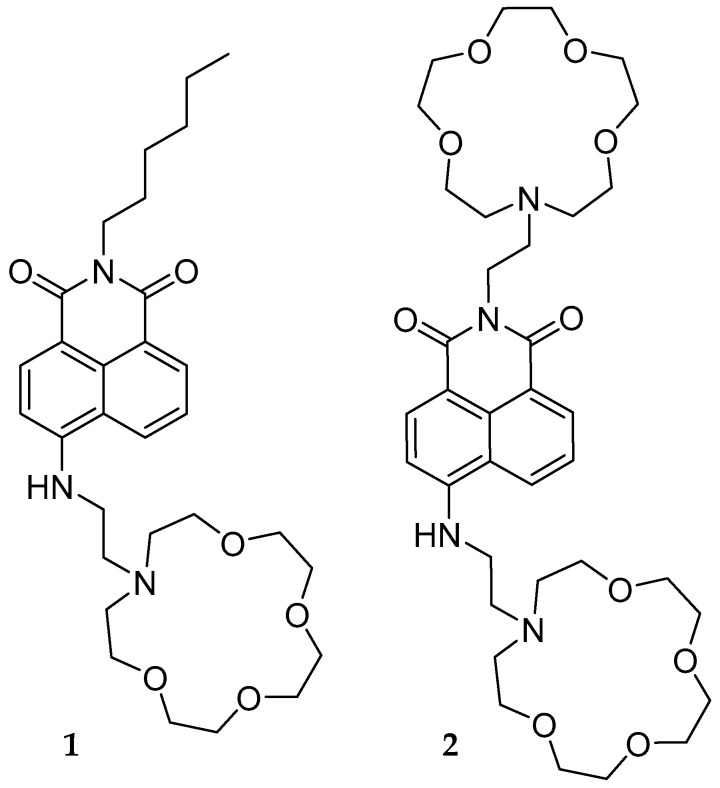
Single- and double-input chemosensors **1** and **2**.

The goal of this study was to incorporate molecular logic gates for the tuning of chemosensor dynamic range. We hypothesized that a two-input AND logic gate with two binding sites for Na^+^ (Figure 1) would display an increased dynamic range compared with a similar chemosensor with only a single Na^+^ receptor. This extension occurs because the AND logic gate with only a single input does not generate a fluorescence response [12]. In particular, every molecule of **1** requires a single Na^+^ ion to generate a fluorescence response, while **2** requires both sites to be occupied to generate the same response (Table 1). Our experiments were conducted as part of W&M’s honors program and supported this hypothesis [13].

## 2. Results

### 2.1. Absorption Spectroscopy

**Figure 2:**
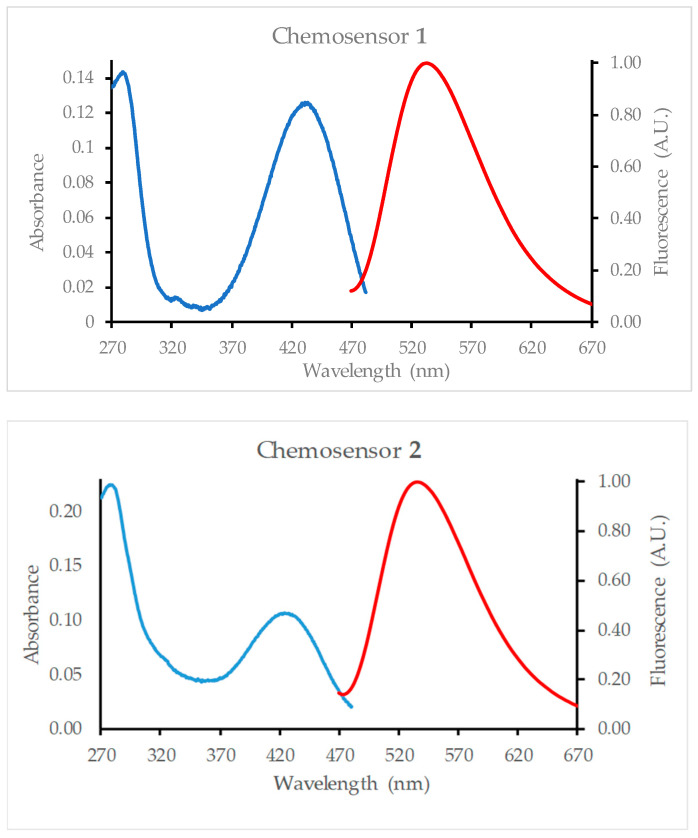
Absorption (**――**) and emission (**――**) spectra of 10 µM methanol solutions of **1** and **2**. Both sensors have an absorption maximum of around 425 nm. Excitation is at 455 nm.

Chemosensor **1** was found to absorb light in the 360–480 nm range with the absorption maximum at 428 nm. Chemosensor **2** absorbs in roughly the same range with an absorption maximum of 424 nm (Figure 2). The molar absorptivity (ε) was calculated as 12,600 M^−1^cm^−1^ and 10,700 M^−1^cm^−1^ for chemosensors **1** and **2**, respectively.

### 2.2. Fluorescence Spectroscopy

Chemosensors **1** and **2** gave a broad emission between 470 nm and 670 nm when excited at 455 nm (Figure 1). The emission maxima were very close: 432 and 436, respectively, for **1** and **2**. The titration of methanol solutions of **1** and **2** with 100 mM aqueous NaCl gave an increase in the fluorescence intensity (Figure 2). At some point, the intensity reached a limiting value and then began to decrease. For chemosensor **1**, saturation was reached around a concentration of 8 mM NaCl, at which point a 4.7-fold increase in fluorescence was observed. Dilution was accounted for when calculating the Na^+^ concentration in the cuvette and the fluorescence intensity. For chemosensor **2**, saturation was reached at 16 mM, with a 3.9-fold increase in the fluorescence intensity.

### 2.3. Binding Constant

**Figure 3:**
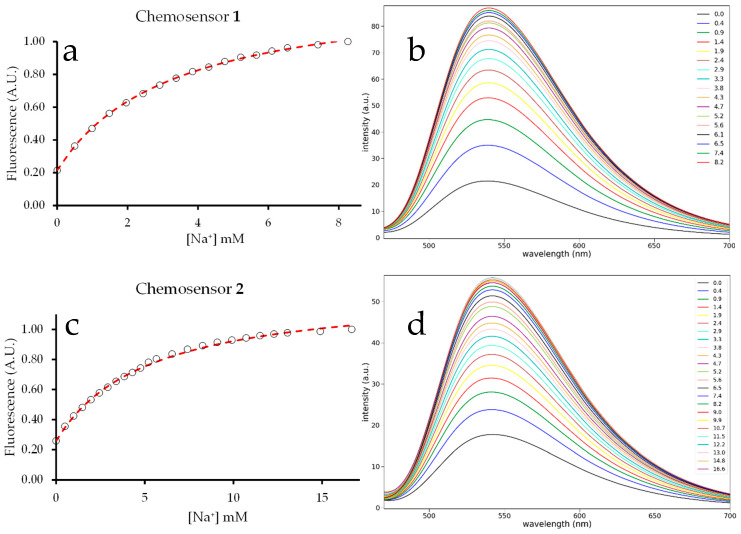
Fluorescence titration plots of chemosensors **1** (**a**,**b**) and **2** (**c**,**d**). Plots (**a**,**c**) are integrated intensity vs. [Na^+^] showing the best fit to Equation (5) (**- - - -**). Plots (**b**,**d**) are the emission spectra at each [Na^+^]. Chemosensors **1** and **2** are 10 µM in methanol. Aliquots of 100 mM aq. NaCl are added to obtain the indicated concentrations. Excitation is at 455 nm.

Using the least squares method, a non-linear curve of best fit was found for both titration plots (Figure 3). After normalizing the fluorescence intensity, a hyperbolic curve was plotted using Equation (5). This equation was derived by relating the measurable change in fluorescence to the fraction of the chemosensor with bound Na^+^ ion [14].

*Fl*: fluorescence intensity at a given [Na^+^]

*Fl_max_*: fluorescence intensity at the saturation point

*Fl*_0_: fluorescence intensity in the absence of Na^+^

[*C*]: free chemosensor concentration

[*A*]: free analyte (Na^+^) concentration

[*C•A*]: concentration of chemosensor bound to the analyte

*k*: binding constant in mM^−1^

*x*: concentration of added analyte (Na^+^) in mM

(1) $$k=\frac{\left[C•A\right]}{\left[C\right][A]}$$

(2) $$bound\;fraction=\frac{Fl-{Fl}_{0}}{{Fl}_{max}-{Fl}_{0}}=\frac{\left[C•A\right]}{\left[C\right]+[C•A]}$$

(3) $$\frac{\left[C•A\right]}{\left[C\right]+[C•A]}\times \frac{\frac{1}{\left[C\right]}}{\frac{1}{\left[C\right]}}=\frac{\frac{\left[C•A\right]}{\left[C\right]}}{\frac{\left[C•A\right]}{\left[C\right]}+1}=\frac{k[A]}{1+k[A]}$$

Equation (3) can be rearranged to isolate measured fluorescence on the left side of the equation.

(4) $$Fl=\frac{\left({Fl}_{max}-{Fl}_{0}\right)k[A]}{1+k[A]}+{Fl}_{0}$$

(5) $$Fl=\frac{\left({Fl}_{max}-{Fl}_{0}\right)kx}{1+kx}+{Fl}_{0}$$

The free analyte concentration [*A*] is the unknown in this equation. The known Na^+^ addition is equal to [*A*] + [*C*•*A*], but it can be approximated as *x*, the concentration of added analyte, since the chemosensor concentration [*C*] is three orders of magnitude smaller than [*A*].

The given constants *Fl_max_* and *Fl*_0_ are known experimentally, while *x* is the varied quantity. The *Fl_max_* value obtained experimentally does not represent the mathematical asymptote of the binding curve, only a lower bound. So, the curve fit algorithm in Python was used to also generate a value for *Fl_max_*. Using these constants, variables, and the titration curve, a curve of best fit can be generated to determine the binding constant (*k*). This process revealed the binding constant for chemosensor **1** to be 0.30 mM^−1^.

The analysis was more difficult for chemosensor **2**, as the two binding sites cannot be titrated in isolation. There are kinetic models that can be used to determine the binding constant when multiple receptors are present; however, the use of such models requires insight into how the fluorescence intensity changes when only a single analyte is bound. If chemosensor **2** behaves as an ideal AND logic gate, the increase in fluorescence intensity is only due to the complex with two Na^+^ ions, one bound in each azacrown ether. On the other hand, molecular logic gates are not always completely binary, as the binding of a single ion can generate a small fluorescence response in an AND logic gate [11].

When the equation above was fitted into the second titration curve, *k* was determined to be 0.19 mM^−1^ from the least squares fit method. This number can be treated as the composite binding constant for the chemosensor, as it is not a measure of the binding constant of either binding site. However, this number is still useful since the equation and the *k* value can be used to calculate the Na^+^ concentration from a fluorescence measurement.

Even though the composite binding constant of **2** is the most useful for our purposes, it is possible to determine the individual binding constants of the receptors on chemosensor **2**. This is achieved by assuming that the crown ether attached to the naphthalene of chemosensor **2** has a binding constant that is equal to that of chemosensor **1**. This model also accounts for the small changes in fluorescence due to the single binding event (Supplementary Information). Assuming a binding constant of 0.31 mM^−1^ for the azacrown in **2** corresponding to **1** gives a binding constant of 0.36 mM^−1^ for the second azacrown in **2**. The product of these two (0.11) is close to the composite binding constant of 0.19 mM^−1^.

Since the titration experiments were conducted with aqueous NaCl, the fluorescence of chemosensor **2** in water and other solvents was examined (Supplementary Information) to ensure that the fluorescence response is due to Na^+^ ion binding and not aggregation-induced emission enhancement (AIEE). Even though the fluorescence of **2** increases significantly as the fraction of water increases in the methanol/water mixture, the increase is not significant below 15% water—the maximum concentration in the NaCl titration—and the emission maximum does not change, even in pure water (Figure S6). In addition, the ion chemoselectivity experiments show that the fluorescence enhancement is due to Na^+^ binding and not AIEE.

### 2.4. Dynamic Range

**Figure 4:**
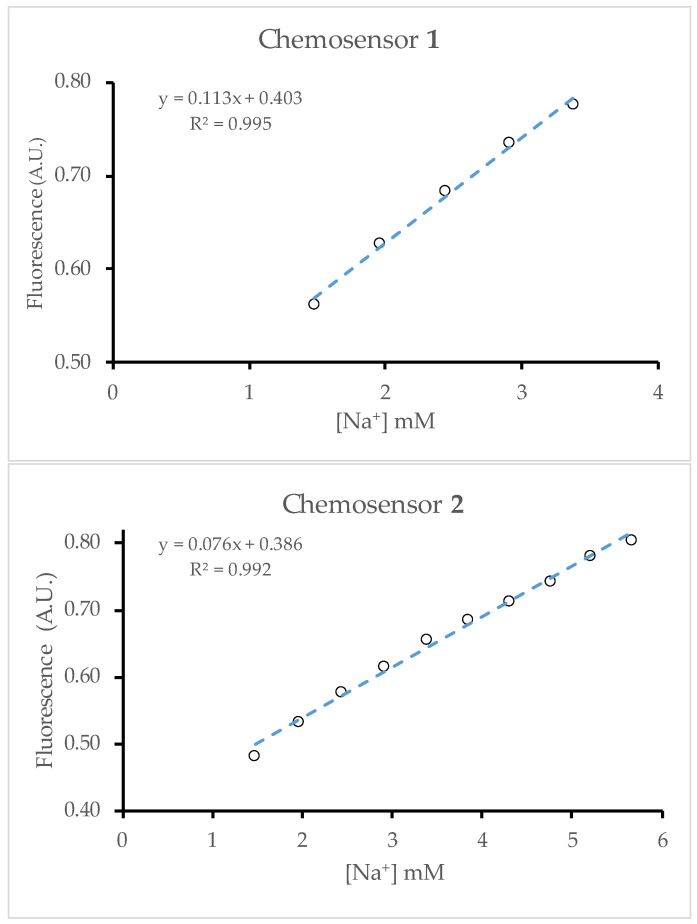
Linear response ranges for **1** and **2** were determined by taking the longest interval in the titration plots with an R^2^ greater than 0.99. The value of R^2^ was calculated by fitting a linear trendline in Excel.

The linear portion of the titration curves was taken as a measure of the dynamic range. Outside the linear range, increases in the analyte concentration will lead to smaller and smaller increases in the fluorescence intensity. Because of this effect, only the linear region of the response curve is useful in quantifying the analyte in each solution. For both plots, a line of best fit was drawn, and R^2^ was kept just above 0.99 [15]. The line of best fit (Figure 4) revealed the detection maximum to be around 3.5 and 6 mM for chemosensors **1** and **2**.

The limits of detection (LoDs) can be approximated using the first part of the titration curve. Here, the response to the addition of sodium ion is the strongest and gives a linear dependence with the points up to 1 mM Na^+^. Linear regression provides the standard error of the y-estimate (σ) and the slope (*m*) from which the limit of detection is calculated to be 3 σ/*m* [16]. This analysis gave LoD values of 0.19 mM for both chemosensors **1** and **2**. When the entire curve is used (Equation (5)), the LoD is 3 σ/*k*. This method gives values of 0.05 and 0.18 mM, respectively. The better value for chemosensor **1** results from the better fit to the data and the larger binding constant.

### 2.5. Chemoselectivity

**Figure 5:**
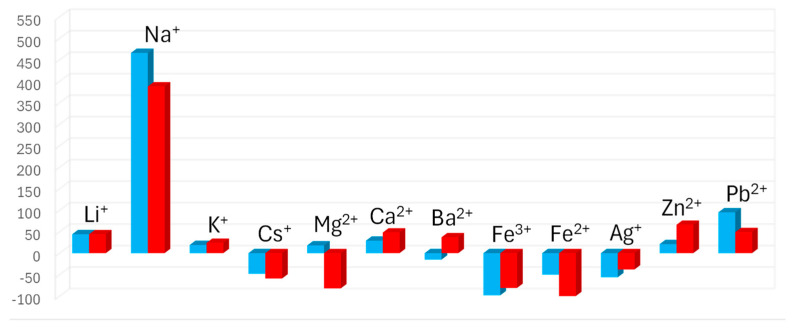
The fluorescence response of chemosensors **1** (blue) and **2** (red) to alkali metals, earth alkali metals, and other transition metals. All cations were added at a concentration of 10 mM to the chemosensors at 10 µM in MeOH. An excitation wavelength of 455 nm was used.

The selectivity of chemosensor **1** with a single 1-aza-15-crown-5 is straightforward. As reported in the literature, Na^+^ ions have the largest affinity towards these crown ethers, while K^+^ ions display a slightly higher affinity compared with other cations [17]. The selectivity of chemosensor **2** is not as straightforward, as molecules with multiple binding sites are known to make sandwich-type complexes with cations [17,18]. More specifically, two 1-aza-15-crown-5 sites are known to bind K^+^ more favorably compared to a single site. For this reason, the fluorescence response of chemosensor **2** with several other cations were tested alongside Na^+^ (Figure 5). No significant change in fluorescence was observed except for in the Na^+^ experiment. The results suggest that the two binding sites on chemosensor **2** are not close enough to form sandwich-type complexes.

## 3. Discussion

Implementing a two-input AND logic gate decreases the binding constant (*k*) from 0.30 mM^−1^ to a composite value of 0.19 mM^−1^. The smaller effective binding constant can be attributed to the fact that chemosensor **2** bound to only a single ion does not display a substantial increase in fluorescence. As a result, the saturation point is increased compared with chemosensor **1**, as the occupation of two binding sites on every molecule of **2** requires twice as many Na^+^ ions. The linear range for **2** was extended to an analyte concentration of ~6 mM compared with ~3.5 mM for **1**.

One of the issues of this study is that the binding sites in **2** are not equivalent. The binding affinity between a receptor and analyte is predominantly determined by the structure of the binding site. Nonetheless, the azacrowns on **2** are attached to the imide and naphthalene parts of the molecule and cannot be assumed to have identical binding affinities towards Na^+^. In the future, this concept can be further tested through the implementation of a symmetric AND logic gate where the binding sites are chemically equivalent.

A second problem is that the AND behavior of chemosensor **2** cannot be directly determined as it is done in a traditional logic gate, where all four input combinations are tested in different experiments [11]. This is not possible in this case since the first and second inputs are the same type of cation, and we cannot study the fluorescence behavior of the singly bound chemosensor **2** in isolation. However, the two-fold extension of the linear range indicates an AND behavior for chemosensor **2**.

**Figure 6:**
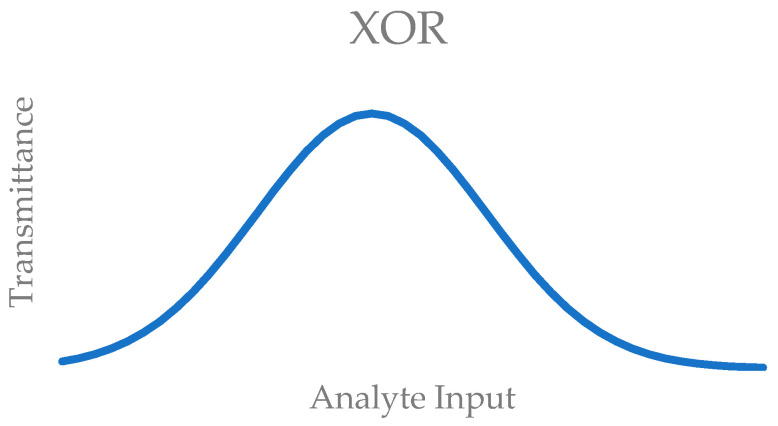
Response curve for an XOR logic gate.

Another future area of study could be the extension of this concept onto other molecular logic gates. For example, an XOR logic gate built in this manner could produce unusual response curves (Figure 6). This logic gate is based on absorbance, and it produces an output when either one of the sites is occupied but not when neither or both sites are occupied [19].

While these implementations are within the realm of possibilities, they are unlikely to be as useful as an AND logic gate. All in all, we were able to extend the dynamic range of a chemosensor without the manipulation of the binding site, while also demonstrating a useful application for molecular logic gates.

## 4. Materials and Methods

To test the hypothesis, chemosensors **1** and **2** were designed and synthesized. The singlet excited state of chemosensor **1** experiences photoinduced electron transfer quenching (PET) from the tertiary amine in the azacrown ether receptor, while chemosensor **2** experiences PET from both receptor amines [20,21]. Due to the fluorescence quenching from both receptors, **2** is expected to behave as an AND logic gate. A 1,8-naphthalimide derivative was chosen as the fluorophore due to its high quantum yield, solubility in various solvents, and various known reactions in the literature [22,23,24]. 1-Aza-15-crown-5 was used as the binding unit due to its chemoselectivity towards Na^+^ [25,26].

### 4.1. Experimental

**Figure 7:**
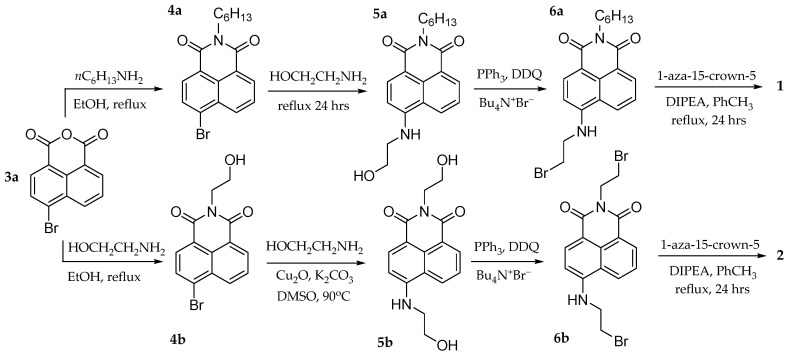
Preparation of chemosensors **1** and **2**.

Reagents were obtained from Acros Organics (Geel, Belgium) or Sigma-Aldrich (St. Louis, MO, USA) unless otherwise in-dicated. Proton and carbon nuclear magnetic resonance spectra were obtained with an Agilent DD2-400 spectrometer (Agilent, Santa Clara, CA, USA). Methanol used for absorption, and fluorescence was spectrophotometric grade. Water was de-ionized by reverse osmosis. Spectrophotometric data were collected using a fiber-optic system with an Ocean Optics Maya CCD detector (Orlando, FL, USA). Absorption spectroscopy used a miniature deuterium/tungsten lamp, and emission spectroscopy used a 455 nm LED light source. Emission intensities were processed by subtracting the electronic noise, converting wavelengths to wavenumbers, multiplying by λ^2^/λ_max_^2^ to account for the effect of the abscissa scale transformation [16], and dividing by the spectral response of the Hamamatsu S10420 CCD. Compounds **3a**–**6a** and **3b**–**6b** are reported molecules in the literature. The general strategy in the synthesis of **1** and **2** was to initially attach the spacer molecules to the fluorophore first, and then the binding site in the final step (Figure 7).

**4a**. Anhydride **3a** (2.00 g, 7.2 mmol) was dissolved in ethanol (50 mL). To this solution, 2 equivalents (1.45 g, 14 mmol) of n-hexylamine was added. The solution was stirred and refluxed for 3 h. The desired product was recrystallized from acetone and filtered to obtain **4a** as a yellow precipitate (2.42 g, 6.7 mmol, 93% yield). The compound was used without further purification [27].

**5a**. Naphthalimide **4a** (0.3 g, 0.83 mmol) was dissolved in ethanolamine (10 mL) and refluxed at 130 °C overnight. The reaction mixture was dissolved in CH_2_Cl_2_ and washed with water twice. The organic layer was dried with MgSO_4_, filtered, and dried under reduced pressure to obtain **5a** as a bright yellow solid (0.27 g, 0.79 mmol, 96% yield).

**6a**. In total, 1.2 equivalents of PPh_3_ (0.25 g, 0.95 mmol) and 2,3-Dichloro-5,6-dicyano-1,4-benzoquinone (DDQ, 0.22 g, 0.95 mmol) were dissolved in CH_2_Cl_2_ (10 mL) and stirred for 15 min. To this solution, 1 equivalent of **5a** (0.27 g, 0.79 mmol) was added. Finally, 1.2 equivalents of tetrabutylammonium bromide (0.31 g, 0.95 mmol) was added, and the solution was stirred at room temperature overnight. The reaction mixture was concentrated in vacuo and purified through column chromatography with pure CH_2_Cl_2_ as the mobile phase to obtain **6a** (0.24 g, 0.60 mmol, 72% yield) [28].

Chemosensor **1**. 1-Aza-15-crown-5 (0.015 g, 0.068 mmol) was dissolved in toluene (5 mL) and stirred under argon gas. **6a** (0.015 g, 0.045 mmol) was dissolved in toluene (5 mL) and added to the mixture. Diisopropylethylamine (DIPEA, 0.032 g, 0.23 mmol) was added, and the mixture was refluxed under argon for 16 h. Then, the toluene was evaporated in vacuo and purified through an automated column chromatography machine (CH_2_Cl_2_:MeOH 0–10%) to obtain chemosensor **1** (0.018 g, 0.032 mmol, 71% yield) [29]. ^1^H NMR (400 MHz, CDCl_3_): 8.57 (d, *J* = 7.5 Hz, 1H), 8.45 (m, 2H), 7.64 (dd, *J* = 7.5, 8.1 Hz, 1H), 7.00 (s, 1H, NH), 6.64 (d, *J* = 8.3 Hz, 1H), 4.14 (m, 2H), 3.64 (m, 16H), 3.40 (m, 2H), 3.00 (m, 2H), 2.85 (m, 4H), 1.71 (m, 2H), 1.45–1.15 (m, 6H), 0.87 (m, 3H); ^13^C {1H} NMR (100 MHZ, CDCl_3_) δ = 163.88, 163.26, 149.12, 133.56, 130.02, 128.89, 126.77, 123.65, 121.73, 119.75, 108.69, 103.31, 69.88, 69.45, 69.00, 68.34, 53.82, 52.66, 39.17, 30.61, 28.68, 27.16, 25.86, 21.57, 13.07. HRMS (ESI): calcd. for C_30_H_44_N_3_O_6_^+^ [M + H]^+^ 542.32246; found 542.322151.

**4b**. Anhydride **3a** (1.00 g, 3.6 mmol) was dissolved in ethanol (50 mL). Ethanolamine (0.55 mL, 4.3 mmol) was added, and the mixture was refluxed for an hour. A white precipitate emerged upon cooling, which was collected by vacuum filtration and washed with water and ethanol to obtain **4b** as a white solid (0.99 g, 3.1 mmol, 86% yield) [30].

**5b**. Naphthalimide **4b** (0.19 g, 0.6 mmol), Cu_2_O (0.17 g, 1.2 mmol), ethanolamine (0.37 g, 6 mmol), and potassium carbonate (0.04 g, 0.3 mmol) were dissolved in DMSO (10 mL). The mixture was heated to 90 °C under argon gas overnight. After cooling to room temperature, the mixture was dissolved in CH_2_Cl_2_ and washed with water three times. The organic layer was vacuum-filtered to remove some of the Cu_2_O, and the filtrate was concentrated. The crude product was purified by silica gel chromatography with EtOAc:MeOH (5:1) as the mobile phase to obtain **5b** (0.13 g, 0.43 mmol, 72% yield) [31].

**6b**. DDQ (0.113 g, 0.5 mmol) and PPh_3_ (0.131 g, 0.5 mmol) were dissolved in CH_2_Cl_2_ (20 mL) and stirred. Then, **5b** (0.030 g, 0.1 mmol) and tetrabutylammonium bromide (0.162 g, 0.5 mmol) were added. The reaction mixture was stirred at room temperature for four hours. The reaction mixture was concentrated under reduced pressure and purified by column chromatography with CH_2_Cl_2_/acetone (9:1) as the mobile phase. Fractions that showed impurities, starting material, and **6b** on TLC were combined and columned again to obtain pure **6b** (0.02 g, 0.047 mmol, 47% yield) [28]. ^1^H NMR (400 MHz, CDCl_3_): 8.63 (d, *J* = 7.2 Hz, 1H), 8.50 (d, *J* = 8.2 Hz, 1H), 8.16 (d, *J* = 8.4, 1H), 7.69 (dd, *J* = 8.2, 7.2 Hz, 1H), 6.77 (d, *J* = 8.4 Hz, 1H), 4.55 (t, *J* = 7.0 Hz, 2H), 3.92 (m, 2H), 3.83 (t, *J* = 7.0 Hz, 2H), 3.76 (t, *J* = 5.9 Hz, 2H). ^13^C {1H} NMR (100 MHZ, CDCl_3_) δ = 164.35, 163.70, 148.55, 134.49, 131.64, 129.89, 126.14, 125.28, 122.92, 120.57, 111.16, 104.68, 44.60, 41.045, 30.85, 28.04

Chemosensor **2**. 1-Aza-15-crown-5 (0.011 g, 0.046 mmol) was dissolved in toluene (10 mL) and stirred under argon gas. **6b** (0.007 g, 0.016 mmol) was added to the mixture. DIPEA (0.022 g, 0.17 mmol) was added, and the mixture was refluxed under argon for 24 h. Then, the toluene was evaporated under reduced pressure and purified through an automated column chromatography machine. A methanol solution with a 10% triethylamine addition was prepared. This mixture was used as the polar solvent in chromatography and was added to CH_2_Cl_2_ with a 0–10% gradient. The fraction with chemosensor 2 was determined to contain triethylammonium salt by ^1^H NMR. The mixture was dissolved in 50 mL of CH_2_Cl_2_ and washed once with 10 mL of water. The organic layer was evaporated to obtain chemosensor **2** (0.0012 g, 0.0017 mmol, 10% yield) [29]. ^1^H NMR (400 MHz, CDCl_3_): 8.55 (d, *J* = 6.9 Hz, 1H), 8.42 (m, 2H), 7.64 (dd, *J* = 7.4, 7.0 Hz, 1H), 7.01 (s, 1H, NH) 6.64 (d, *J* = 6.9 Hz, 1H), 4.22 (m, 2H), 3.65 (m, 34H), 3.39 (m, 2H), 2.98–2.79 (m, 10H); ^13^C {1H} NMR (100 MHZ, CDCl_3_) δ = 164.85, 164.18, 150.29, 134.66, 131.03, 129.95, 127.87, 124.62, 122.62, 120.76, 109.42, 104.38, 70.89, 70.84, 70.45, 70.41, 70.33, 70.20, 70.13, 69.96, 69.80, 69.55, 54.94, 54.87, 53.57, 40.59, 37.59, 29.69; HRMS (ESI): calcd. for C_36_H_55_N_4_O_10_^+^ [M + H]^+^ 703.39127; found 703.39124.

The NMR spectra for compounds **3a**–**6a** and **3b**–**5b** are reported in the literature as well [28,31,32,33,34].

### 4.2. Spectroscopy

Chemosensors **1** and **2** were diluted with 50 and 25 mL of spectroscopic-grade methanol, respectively, to obtain 0.64 and 0.068 mM solutions. A total of 156 and 1470 µL of these solutions were diluted to 10 mL to obtain 10 µM methanol solutions of each chemosensor. Stock solutions of NaCl in water in 1, 10, and 100 mM concentrations were prepared.

For absorption spectroscopy, measurements were taken as an average of 25 scans, each taking 160 ms on the Maya spectrometer. For chemosensors **1** and **2**, three different samples were measured: dark, blank, and the chemosensor itself. I_0_ and I in this instance are obtained by subtracting the dark measurement from the blank and chemosensor sample to account for background noise. The transmittance was measured for the three samples between 200 and 1100 nm, and the Beer-Lambert was used to obtain the absorbance.

With consideration of the absorption spectra, 455 nm was selected as the excitation wavelength for the fluorimetry experiments. Ten scans were averaged for each measurement, with an integration time of 400 ms. Dark scans were taken for each experiment to account for the background noise. The reported fluorescence intensities are the normalized integrated values from 14,000 cm^−1^ to 21,000 cm^−1^.

## Figures and Tables

**Table 1:** Truth table for an AND logic gate. Four different combinations of input are shown on the left two columns. An output is generated when both inputs are present.

Input I	Input II	Output
0	0	0
1	0	0
0	1	0
1	1	1

## Data Availability

The data presented in this study are available either in this article or in the Supplementary Materials.

## Supplementary Materials

The following supporting information can be downloaded at https://www.mdpi.com/article/10.3390/molecules29184330/s1: Figures S1–S3: Proton and carbon NMRs for compounds **1**, **2** and **6b**; Figures S4 and S5: Mass spectrum of compounds **1** and **2**; Binding analysis with non-equivalent azacrowns; Figure S6: Fluorescence spectra of **2** in various solvents; Figure S7: Plot of emission center-of-gravity for 2 vs. *E*_T_(30).
