# Supplementary material for: Use of Molecular Logic Gates for the Tuning of Chemosensor Dynamic Range
Source: Molecules. 2024 Sep 12;29(18):4330. doi: 10.3390/molecules29184330 (PMC11434483; doi:10.3390/molecules29184330)
Supplement: Supplementary file 1 [file molecules-29-04330-s001.zip › molecules-3081564-supplementary.pdf]

Use of Molecular Logic Gates for The Tuning of Chemosensor Dynamic Range

Orhan Acikgoz [1,*] and Christopher Abelt [2]

Supplementary Materials

Table of Contents

Title and Table of Contents S1
NMR spectroscopy S2
Figure S1. ^{1}H and ^{13}CNMR spectra of **1**. S2
Figure S2. ^{1}H and ^{13}CNMR spectra of **2** S3
Figure S3. ^{1}H and ^{13}CNMR spectra of **6b** S4
Mass Spectrometry S5
Figure S4. HRMS spectrum of **1** S5
Figure S5. HRMS spectrum of **2** S6
Binding analysis with non-equivalent azacrowns S7
Fluorescence spectroscopy S8
Figure S6. Fluorescence spectra of **2** in various solvents S8
Figure S7. Plot of emission center-of-gravity for 2 vs. $E_T(30)$ S8

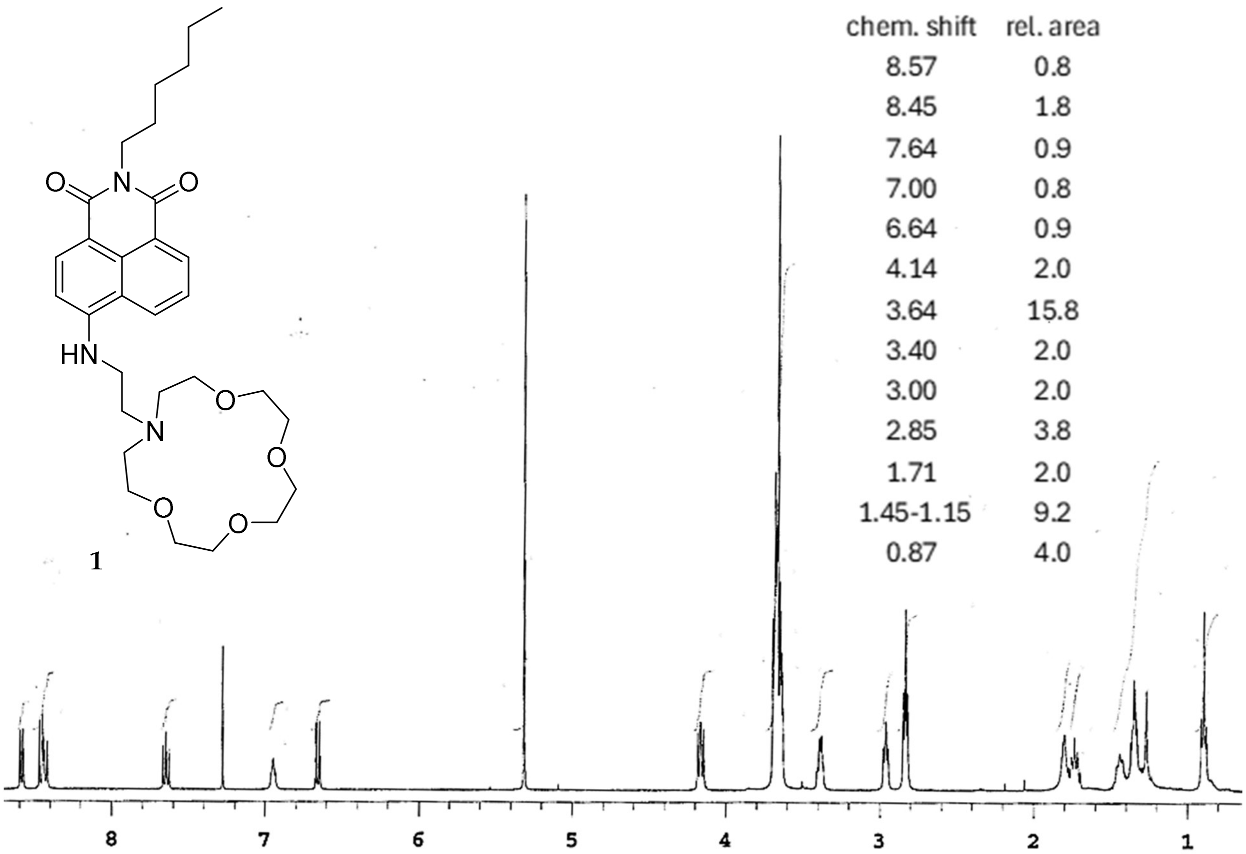

1

chem. shift	rel. area
8.57	0.8
8.45	1.8
7.64	0.9
7.00	0.8
6.64	0.9
4.14	2.0
3.64	15.8
3.40	2.0
3.00	2.0
2.85	3.8
1.71	2.0
1.45-1.15	9.2
0.87	4.0

chem. shift		
163.88	121.73	53.82
163.26	119.75	52.66
149.12	108.69	39.17
133.56	103.31	30.61
130.02	69.88	28.68
128.89	69.45	27.16
126.77	69.00	25.86
123.65	68.34	21.57
		13.07

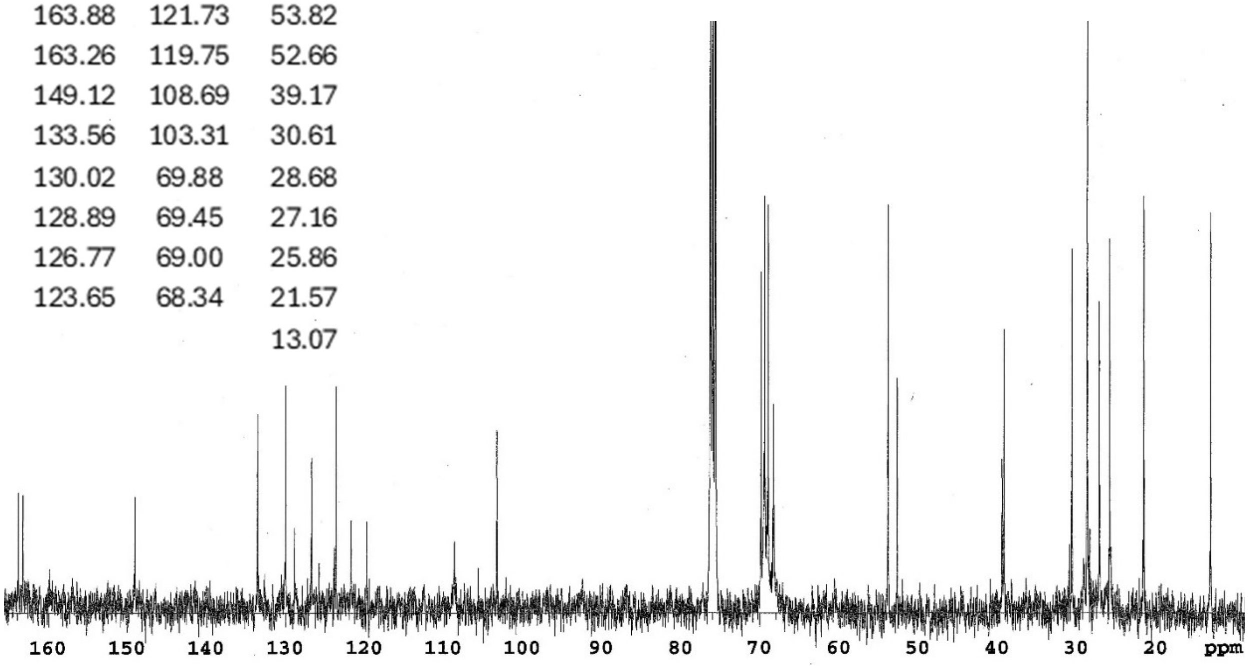

Figure S1. ^{1}H and ^{13}CNMR spectra of **1**.

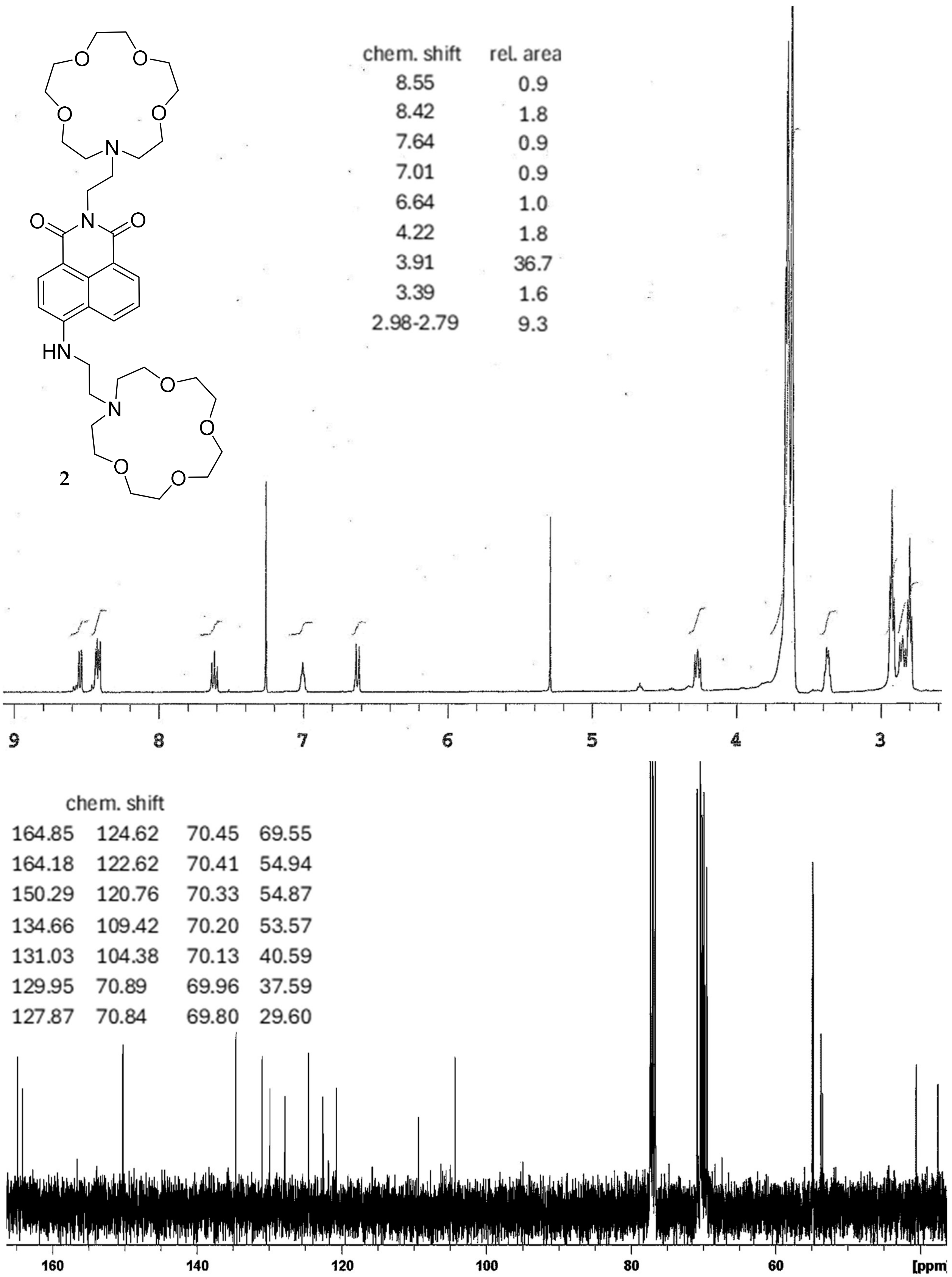

Figure S2. ^{1}H and ^{13}CNMR spectra of **2**.

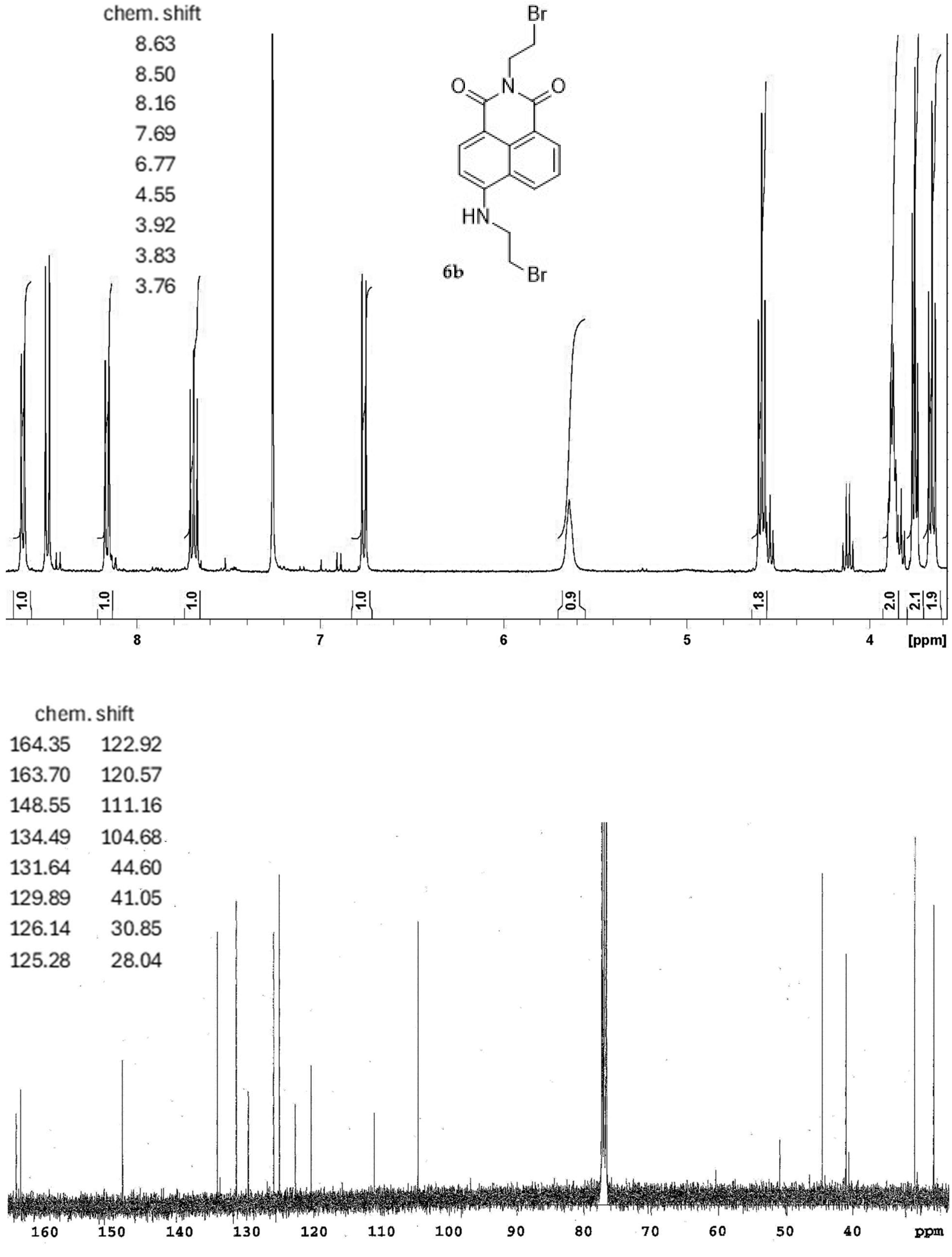

Figure S3. ^{1}H and ^{13}CNMR spectra of **6b**.

Mass Spectrum Lst Report

**Analysis Info**			Acquisition Date	4/4/2024 9:39:31 AM
Analysis Name	D:\MS1 COSMIC\2024\apexdata040424\orhan1_pos_000002.d			
Method			Operator	COSMIC
Sample Name	orhan1		Instrument	apex-Qe
Comment	orhan1 in MeOH	C30H43N3O6 H+		

Sample Name	orhan1 in MeOH			
Exact Mass of	C30H43N3O6 H+	=	542.322463	m/z
Mass Observed		=	542.322151	m/z

Difference < 1.0 ppm

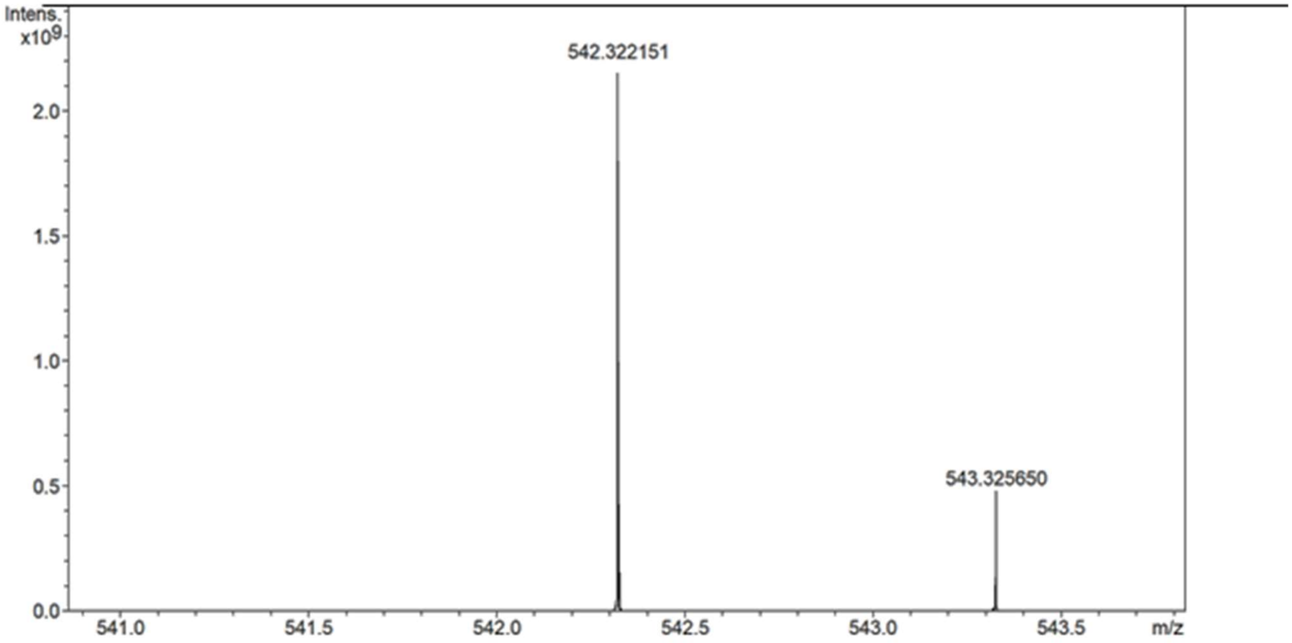

#	m/z	I
1	187.5897	65607016
2	467.255	32249274
3	542.3168	23411550
4	542.3185	33233758
5	542.3222	2193136384
6	542.3237	144087904
7	542.3245	58858336
8	542.3253	32578398
9	543.3257	484088704
10	543.3272	30219122
11	544.3298	54975368
12	564.3044	869047488
13	565.308	271555744
14	566.3122	31521970
15	580.2788	156048640
16	581.2828	44227844

Figure S4. HRMS spectrum of **1** listing all ions with an abundance greater than 1% of the base.

Mass Spectrum List Report

**Analysis Info**		Acquisition Date	4/4/2024 10:25:26 AM
Analysis Name	D:\MS1 COSMIC\2024\apexdata040424\orhan2_pos_000003.d		
Method		Operator	COSMIC
Sample Name	orhan2	Instrument	apex-Qe
Comment	orhan2 in MeOH:THF C36H54N4O10 H+		

Sample Name orhan2 in MeOH:THF

Exact Mass of C36H54N4O10 H+ = 703.391270 m/z

Mass Observed = 703.391244 m/z

Difference < 1.0 ppm

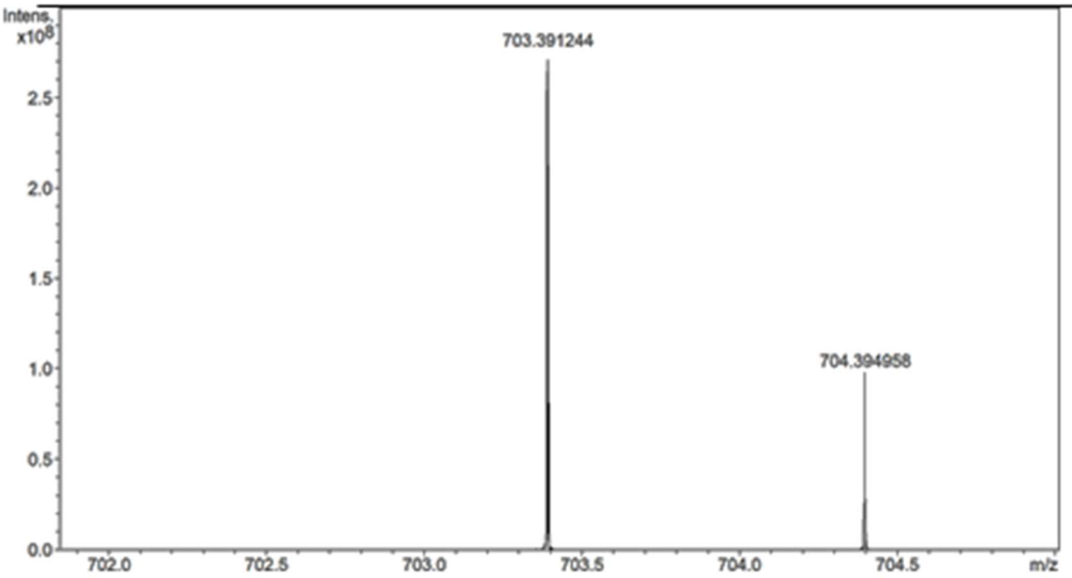

#	m/z	I
1	480.211081	16090945
2	487.263193	12601179
3	520.221668	10387534
4	542.203585	24263002
5	564.305208	17267332
6	677.377024	10391347
7	703.391244	276079168
8	703.393742	16073280
9	704.394958	98345544
10	725.372577	983343616
11	725.377572	16384539
12	725.37899	10629659
13	726.376221	354329088
14	727.353569	10650115
15	727.379884	61497860
16	803.28487	37511560
17	805.283362	35004812

Bruker Compass DataAnalysis 4.0   printed: 4/4/2024 10:33:16 AM

Figure S5. HRMS spectrum of **2** listing all ions with an abundance greater than 1% of the base.

Binding analysis with non-equivalent azacrowns

Fl:	fluorescence intensity at a given [Na+]
Fl_{00}:	fluorescence intensity in the absence of Na+
Fl_{10}:	fluorescence intensity with top crown complexed
Fl_{01}:	fluorescence intensity with bottom crown complexed
Fl_{11}:	fluorescence intensity with both crowns complexed
[*C*]:	free chemosensor concentration
[*A*]:	free analyte (Na^+) concentration
[*C•A*]:	concentration of chemosensor bound to the analyte
k:	binding constant in mM^{-1}
x:	concentration of added analyte (Na^+) in mM

In a more detailed analysis there are four fluorescent species shown below. The fluorescence of complex **10** is assumed to be the same as the uncomplexed chemosensor because it has the same amino group available for PET. The binding constant for the formation of complex **01** is assumed to be the same as chemosensor **1** (0.31 mM^{-1}). The binding constant for the formation of the double complex is approximated as the product of the two single binding constants ($k_{11} = k_{10} \times k_{01}$). Curve-fitting of the equation below provides values for Fl_{01}-Fl_{00}, Fl_{11}-Fl_{00}, and k_{10}: 0.933, 0.524, and 0.36. The curve-fit is shown below.

$$Fl - Fl_{00} = \frac{(Fl_{01} - Fl_{00})k_{01}x + (Fl_{11} - Fl_{00})k_{11}x^2}{1 + k_{01}x + k_{10}x + k_{11}x^2}$$

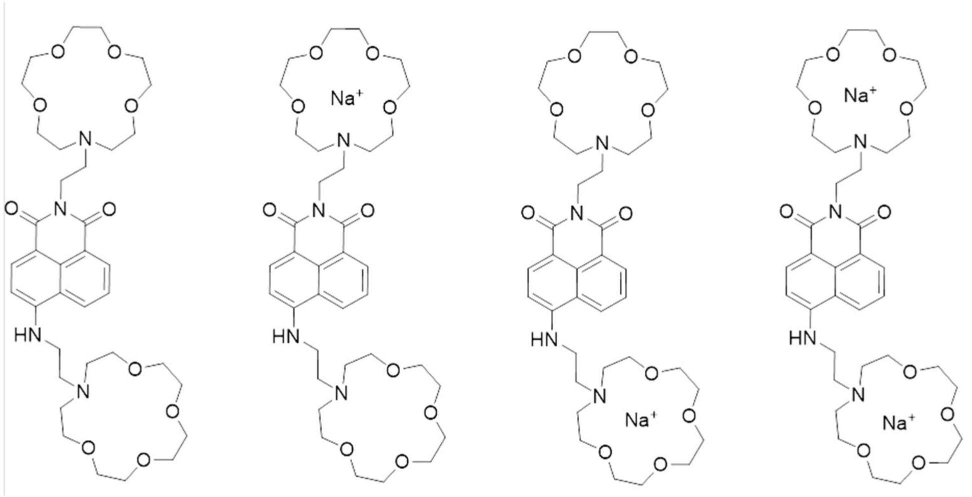

00 10 01 11

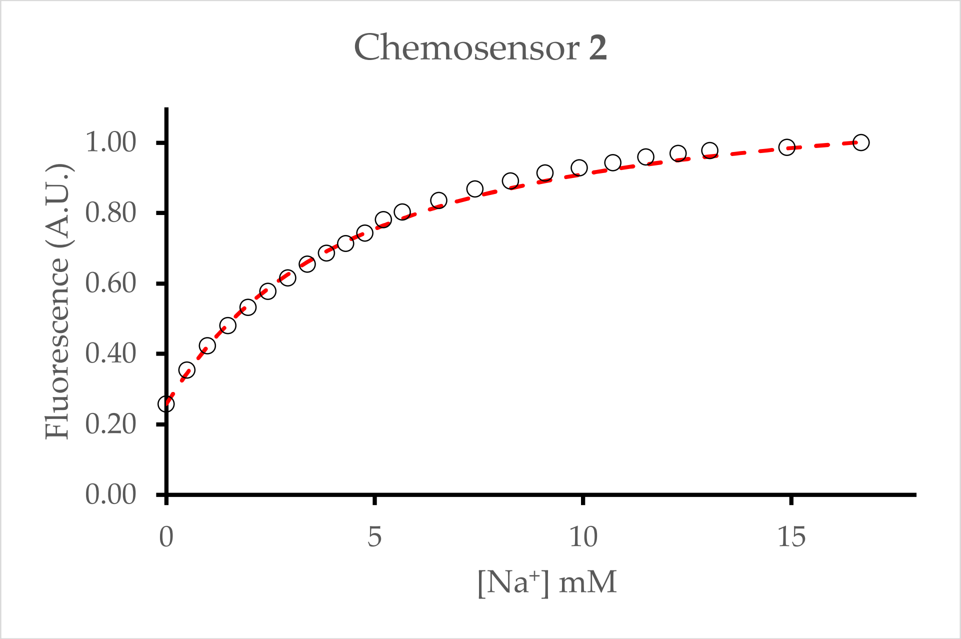
Chemosensor 2
Fluorescence (A.U.)
1.00
0.80
0.60
0.40
0.20
0.00
0
5
10
15
[Na+] mM

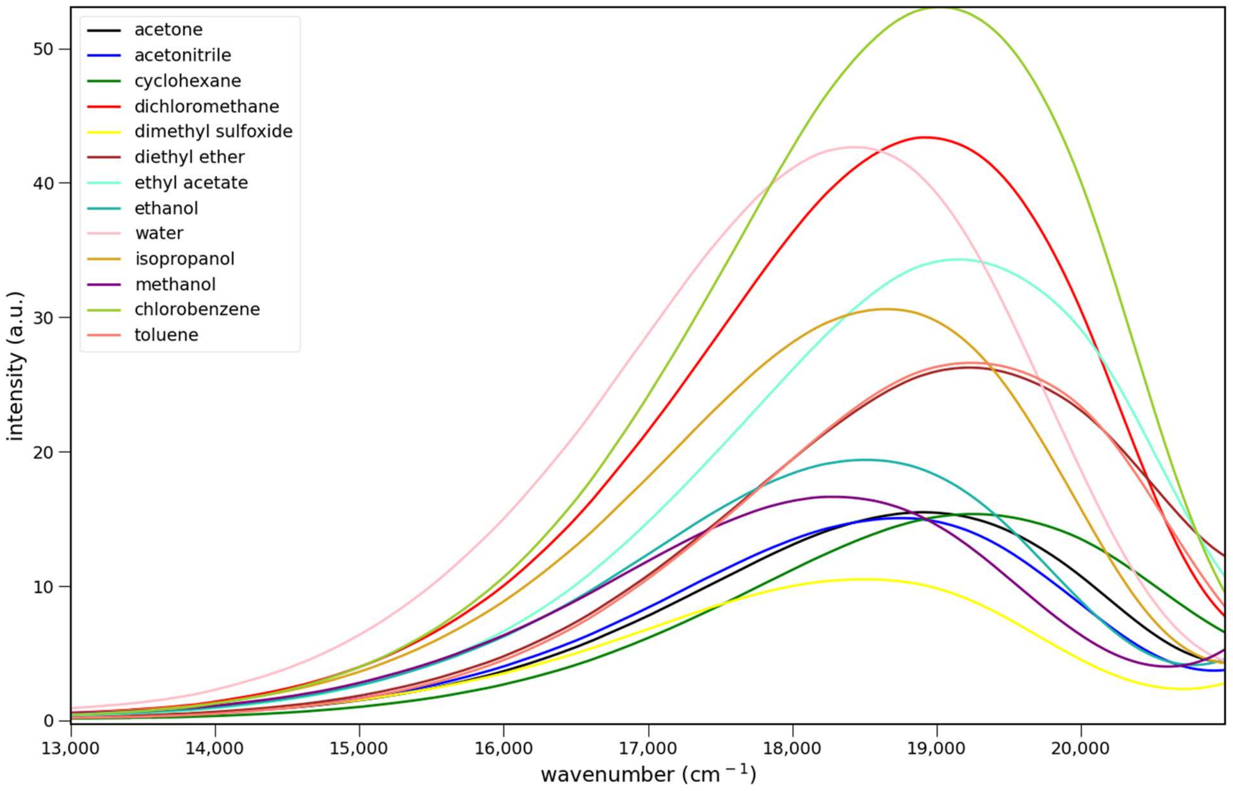

Figure S6. Fluorescence spectra of 10 μM **2** in various solvents. Excitation at 455 nm.

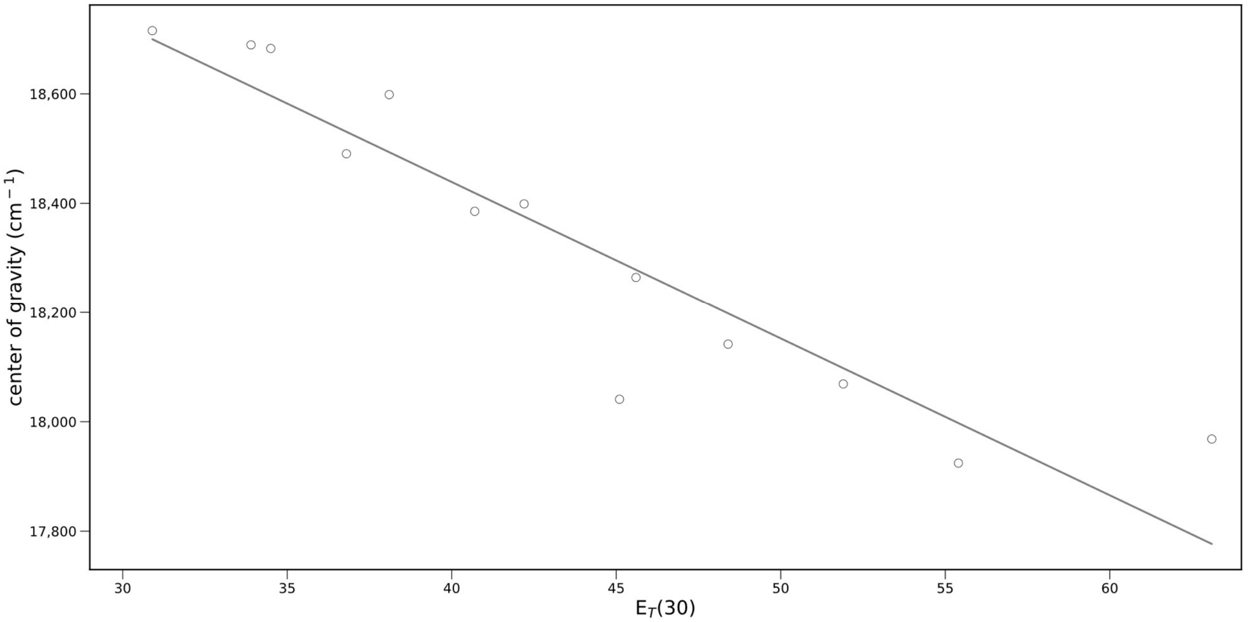

Figure S7. Plot of the emission center-of-gravity (cm^{-1}) vs. $E_T(30)$ for **2**. Excitation at 455 nm.
